# Long-term effect of azithromycin in bronchiolitis obliterans syndrome

**DOI:** 10.1136/bmjresp-2019-000465

**Published:** 2019-10-15

**Authors:** C.Tji-Joong Gan, Chris Ward, Gerard Meachery, James Laurence Lordan, Andrew J Fisher, Paul A Corris

**Affiliations:** 1 Pulmonary Diseases, University Medical Centre Groningen Thoraxcentre, Groningen, The Netherlands; 2 Transplantation Institute of Cellular Medicine, Newcastle University, Newcastle upon Tyne, UK; 3 Respiratory Medicine, Freeman Hospital, Newcastle upon Tyne, UK

**Keywords:** lung transplantation

## Abstract

**Introduction:**

Azithromycin stabilises and improves lung function forced expiratory volume in one second (FEV_1_) in lung transplantation patients with bronchiolitis obliterans syndrome (BOS). A post hoc analysis was performed to assess the long-term effect of azithromycin on FEV_1_, BOS progression and survival .

**Methods:**

Eligible patients recruited for the initial randomised placebo-controlled trial received open-label azithromycin after 3 months and were followed up until 6 years after inclusion (n=45) to assess FEV_1_, BOS free progression and overall survival.

**Results:**

FEV_1_ in the placebo group improved after open-label azithromycin and was comparable with the treatment group by 6 months. FEV_1_ decreased after 1 and 5 years and was not different between groups. Patients (n=18) with rapid progression of BOS underwent total lymphoid irradiation (TLI). Progression-free survival (log-rank test p=0.40) and overall survival (log-rank test p=0.28) were comparable. Survival of patients with early BOS was similar to late-onset BOS (log-rank test p=0.74).

**Discussion:**

Long-term treatment with azithromycin slows down the progression of BOS, although the effect of TLI may affect the observed attenuation of FEV_1_ decline. BOS progression and long-term survival were not affected by randomisation to the placebo group, given the early cross-over to azithromycin and possibly due to TLI in case of further progression. Performing randomised placebo-controlled trials in lung transplantation patients with BOS with a blinded trial duration is feasible, effective and safe.

**Key messages**

- Placebo-controlled trials in patients with bronchiolitis obliterans syndrome (BOS) are scarce, and long-term outcomes of such trials are generally lacking in lung transplantation.

- Long-term treatment with azithromycin slows down the progression of BOS; BOS progression and long-term survival are not affected by randomisation to the placebo group.

- This post hoc analysis strengthens the evidence of long-term azithromycin treatment in BOS and shows the validity and ethical appropriateness of conducting placebo-controlled trials in this difficult area.

## Introduction

Lung transplantation is a preferred option in selected patients with end-stage lung disease that continues to progress.^1^ Despite improvement in quality of life, long-term survival remains poor due to the development of chronic lung allograft dysfunction, including bronchiolitis obliterans syndrome (BOS). Within 5 years, approximately 50% of the lung transplant recipients developed BOS.^2^ Previous studies have shown that azithromycin can stabilise and improve lung function in lung transplantation recipients with BOS.^3–11^ In addition, only one placebo-controlled trial of azithromycin in lung transplant recipients was performed with a positive effect on lung function after 3 months.^12^ A post hoc analysis was performed to assess the long-term effect on the evolution of lung function, progression of BOS and survival in patients initially randomised to placebo or azithromycin.

## Methods

### Study population

**Figure 1:**
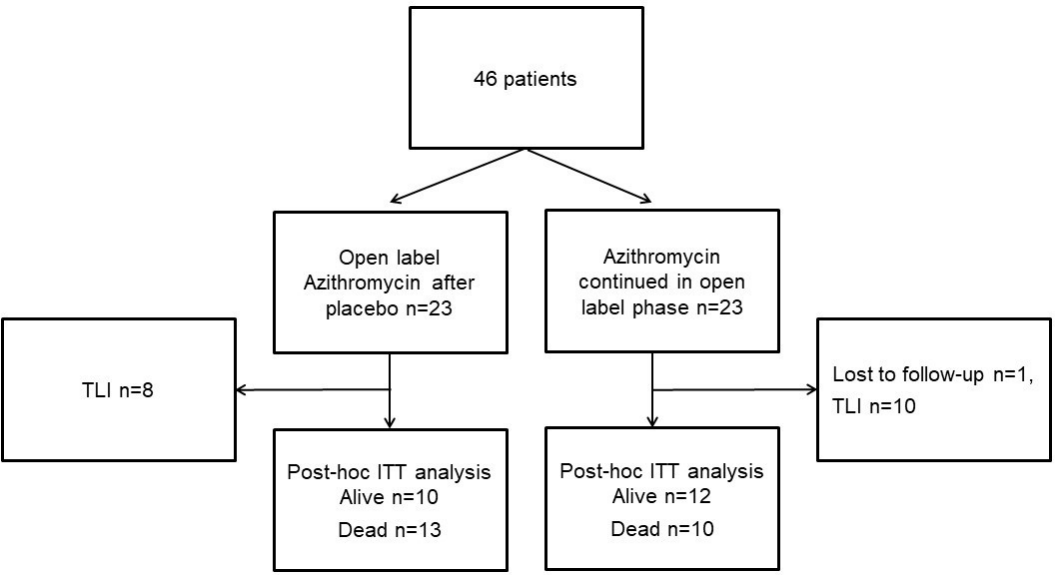
Flowchart of the placebo (n=23) and azithromycin (n=23) groups, with a follow-up period of at least 6 years after the inclusion of the last patient. Patients were randomised and initially evaluated after 3 months. One patient was lost to follow-up. Eighteen patients underwent TLI because of rapid progression of forced expiratory volume in one second decline. ITT, intention to treat; TLI, total lymphoid irradiation.

Patients were initially included for the double-blind, randomised placebo (n=23) controlled trial with azithromycin 250 mg (n=23) on alternate days (EU-CTR, 2006-000485-36/GB) between November 2006 and December 2010 from the Freeman Hospital NHS, Newcastle upon Tyne, UK. Inclusion criteria were male or female lung transplant recipients over the age of 16 years, airflow limitation–forced expiratory volume in one second (FEV_1_) <80% maximum recorded post-transplant (BOS grades 1–3) and ability to give informed consent. Exclusion criteria were airflow limitation caused by other clinical problems (eg, clinical infection), known hypersensitivity to azithromycin or any of the macrolide antibiotics, participation in another drug-related trial within 90 days, severe renal or hepatic impairment and use of concomitant medication that would interact with azithromycin. After the study period of 12 weeks, all patients received open-label azithromycin. Patients were followed up in the outpatient clinic on a regular basis (3–4 months) with lung function, FEV_1_ and forced vital capacity (FVC) until 6 years after inclusion of the last patient. There was one patient lost to follow-up originally allocated to the azithromycin group. Data of 45 patients were available for post hoc analysis. A flowchart is shown in figure 1.

Lung function was assessed and compared after 6 months, 1 and 5 years between the treatment and placebo groups. Progression of BOS, defined as >10% drop in FEV_1_ over time, and mortality were retrospectively analysed according to an intention-to-treat approach comparing the placebo and azithromycin groups from the initial randomised trial.

All patients received standard triple immunosuppression with calcineurin inhibitor (cyclosporine A or tacrolimus), a cell cycle inhibitor (azathioprine or mycophenolate mofetil) and prednisolone 0.1 mg/kg. Patients with rapid progression of graft dysfunction despite azithromycin during the open-label phase, defined as a persistent or progressive decrease in FEV_1_ of 0.5 mL or more between two visits at the outpatient clinic, were referred for total lymphoid irradiation (TLI). All patients provided written informed consent before randomisation. The institutional review board approved the study.

### Statistical analysis

GraphPad Prism V.8.0 software (San Diego, CA USA) was used for statistical analyses, all performed on an intention-to-treat basis by comparing patients assigned in the initial study to the placebo and azithromycin groups. Data are shown as median with IQR. Two-way analysis of variance was used to analyse the evolution of lung function. Freedom from progression of BOS, defined as >10% drop in FEV_1_ at follow-up or referral for TLI, survival and the effect of BOS severity and time to BOS were assessed by construction of Kaplan-Meier curves and log-rank test. A p value of <0.05 was regarded as statistically significant.

Patient and public involvement statement: not involved.

## Results

### Patient characteristics

Data of 45 patients were available for analysis. Follow-up time was not different between the groups. (azithromycin 11.0±4.2 years vs placebo 9.0±4.2 years). Table 1 summarises the main patient characteristics. The median age at the time of transplantation was 47 years. The majority of patients had emphysema as pretransplant diagnosis (42%), and 60% of the patient group underwent a bilateral lung transplantation. The median time to the diagnosis of BOS was 2.6 years (1.36–6.95). From the total study group, 60% had BOS grade 1, and 40% had BOS grade 2 or 3. During the follow-up period, there were no side effects reported related to azithromycin.

**Table 1:** Demographic and clinical characteristics

Subjects	n=45
Age at LTx	47 (52–37)
Sex (M/F)	24/21
Pretransplant diagnosis	
Emphysema	19 (42%)
CF	12 (27%)
PF	6 (13%)
Other	8 (18%)
Tx procedure	
Single	17 (38%)
Double	27 (60%)
Heart–lung	1 (2%)
FEV_1_ (L)	1.6 (1.2–2.2)
FVC (L)	2.83 (2.21–3.52)
Time to BOS (years)	2.6 (1.36–6.95)
BOS grade	
1	27 (60%)
>1	18 (40%)

Median (IQR).

BOS, bronchiolitis obliterans syndrome; CF, cystic fibrosis; F, female; FEV_1_, forced expiratory volume in one second; FVC, forced vital capacity; LTx, Lung transplantation; M, male; PF, pulmonary fibrosis; Tx, Transplantation.

### Evolution of lung function

**Figure 2:**
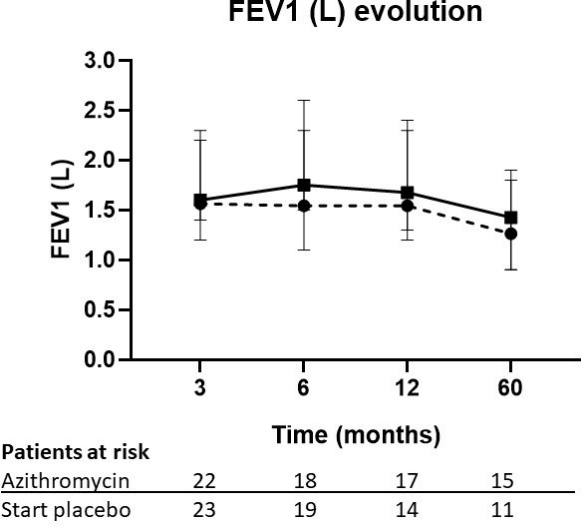
Evolution of FEV_1_ in litre in the placebo+azithromycin after 3 months group (solid line) and the azithromycin group (dashed line). FEV_1_, forced expired volume in one second.

Figure 2 shows the evolution of FEV_1_ litres in the patient groups after open-label azithromycin. After 6 months, FEV_1_ of the initial placebo group increases but is not different from the patients randomised to the azithromycin group (placebo: 1.7 L (1.5–2.6) vs azithromycin: 1.5 L (1.1–2.3), p=not significant (NS)). After 1 year (placebo; 1.3 L (0.9–1.8) vs azithromycin; 1.5 L (1.2–2.3), p=NS) and 5 years, FEV_1_ decreases gradually (placebo: 1.4 L (0.9–1.9) vs azithromycin: 1.6 L (1.3–2.4), p=NS) but is not significantly different between the groups.

### Progression of BOS

**Figure 3:**
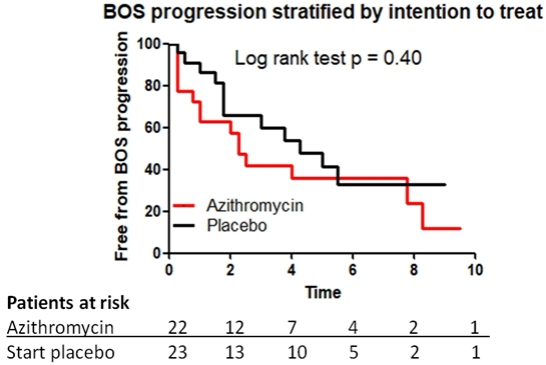
Freedom from BOS progression, >10% decline in forced expired volume in one second, in patients started with placebo (black) group and in patients in the azithromycin (red) group. BOS, bronchiolitis obliterans syndrome.

After the start of the randomised trial, four patients randomised to placebo received rescue azithromycin because of rapid deterioration as per protocol, and one patient withdrew consent.^12^ In total, 18 patients underwent TLI therapy because of further clinical deterioration and a drop in FEV_1_ (8 patients in the placebo group and 10 patients in the azithromycin randomised group). The time from start of azithromycin to TLI was 7.7 months (0.3–36.5) and was not different between the groups. Figure 3 shows no difference in time to progression of graft dysfunction between the azithromycin and placebo groups.

### Survival analyses

**Figure 4:**
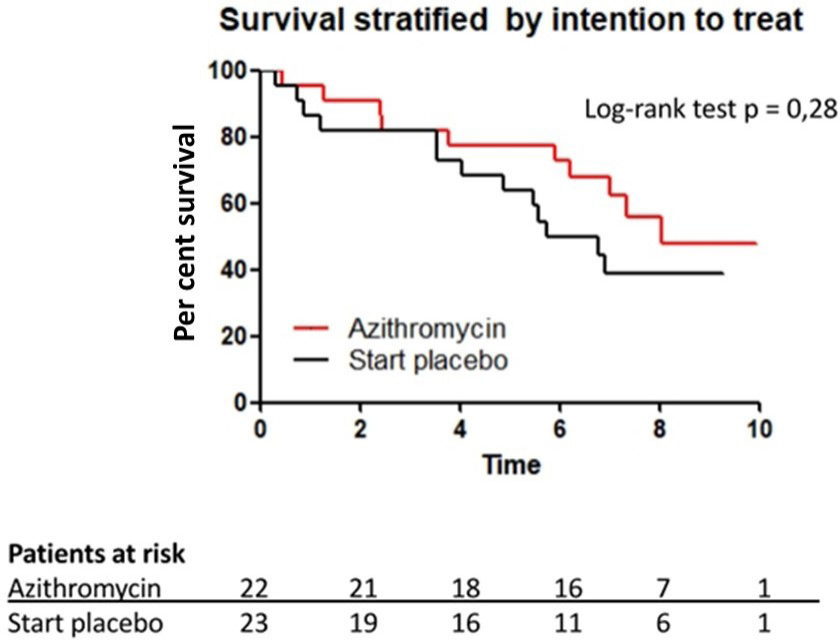
Post hoc intention-to-treat survival analysis in patients started with placebo group (black) and in patients in the azithromycin group (red).

**Figure 5:**
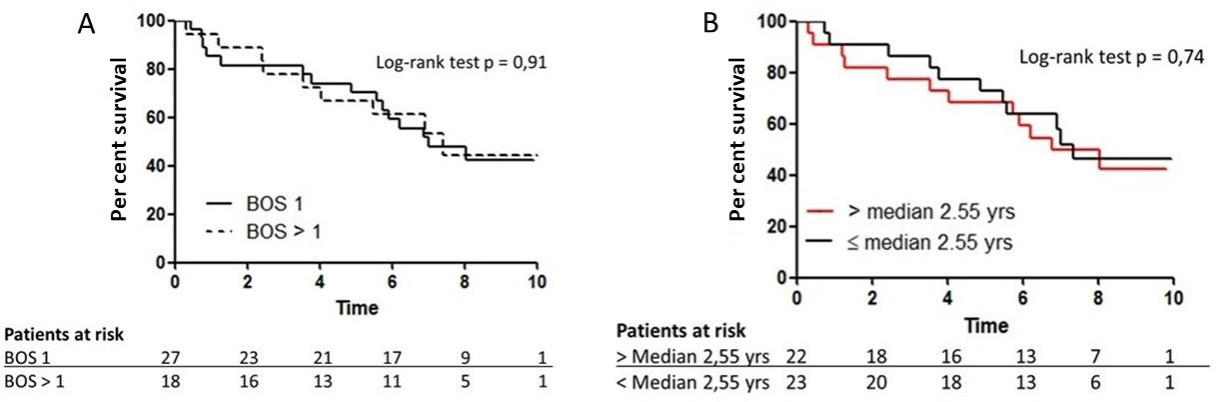
(A) Post hoc intention-to-treat survival analysis stratified by BOS severity: BOS grade 1 (solid line) and BOS grade >1 (dashed line) (B). Post hoc intention-to-treat survival analysis stratified by time to onset of BOS. BOS, bronchiolitis obliterans syndrome.

During the follow-up period, 24 patients died as a consequence of respiratory failure or infections. There was no effect of initial randomisation on survival (figure 4). In addition, 5-year survival after the onset of BOS was 77% in the azithromycin group and 64% in the placebo group (log-rank test p=0.28). Furthermore, BOS severity had no effect on survival (figure 5A). Five-year survival was 71% in patients with BOS 1 and 67% in patients with a higher grade than BOS 1. Patients with early onset of BOS (≤median 2.6 years) had similar survival as patients with late onset of BOS (>median 2.6 years) (figure 5B); 5-year survival was 68% and 73%, respectively.

## Discussion

This work represents the first post hoc analysis of the long-term effect of azithromycin in BOS of a double-blind placebo-controlled trial in lung transplantation patients. Compared with the group initially treated with azithromycin, the lung function of patients on placebo stabilises after starting azithromycin. Progression-free survival and overall survival were not different between the groups. In addition, in this group, BOS severity and the time to development of BOS did not affect survival. This analysis shows that conducting randomised placebo-controlled trials is safe in terms of progression of BOS and long-term survival.

A recent meta-analysis by Kingah *et al* 13 demonstrated that there is an improvement of lung function in patients with BOS after 7 months. The majority of studies included were retrospective. The only randomised placebo-controlled trial from our centre showed an improvement in FEV_1_ with azithromycin.^12^ The natural course of BOS in lung transplantation has been reported by Lama *et al*.^14^ In this observational study, there was a steep decline in FEV_1_ in the first 6 months. The data in our study showed that in the treatment group, lung function stabilises, and in the placebo group, after an initial decline, lung function improves after 3 months of open-label treatment with azithromycin to a level similar to the treatment group. Although the lung function decline is progressive over time in our study group, this is a gradual process. In the study by Lama *et al*,^14^ average lung function decrease was approximately 16% in the first year after onset of BOS; however, BOS was less severe in our study group. Compared with the Lama *et al* data, there was no progressive decline in lung function with azithromycin in our study group in the first year after BOS onset, and in agreement with the observational study by Shitrit *et al*,^8^ azithromycin slowed down progression of BOS within the first year of BOS onset. After 5 years, there was a further decline of FEV_1_; however, an approximately 0.30 mL (10%) decline in FEV_1_ in 4 years with azithromycin seems a clinical significant finding and supports the positive impact of azithromycin.

Progression of BOS, arbitrarily defined as >10% decline of FEV_1_ or referral for TLI, was not different between the groups. This might suggest that performing a randomised placebo-controlled trial is safe in terms of progression of BOS. In addition, long-term survival was not different between the groups. Our study showed a better 5-year survival when compared with the overall survival in the study by Finlen Copeland *et al*.^15^ More intensive immunosuppression after the onset of BOS may have resulted in higher risk of infections and death consequently. However, in the Copeland study, there was no relation between intensive immunosuppression and survival. In addition, in our study population, more intensive immunosuppression was not a common practice. Time to BOS onset and BOS severity affected survival in the Copeland study but were not significant in our study. The median onset time was shorter in our study and may have resulted in earlier participation to the randomised placebo-controlled trial and thus intervention with azithromycin. This is supported by the Vos *et al* study, where earlier initiation of azithromycin was protective of BOS progression and death.^11^ Forty per cent of our study population had higher grades of BOS (>1) compared with the Copeland study. During the clinical trial period, but also on regular clinical visits, patients with higher grades of BOS are followed up more closely, and there is a low threshold to treat acute rejection and infections. Our long-term follow-up data corroborate with the study findings of Ruttens *et al*.^16^ In their study, prophylactic azithromycin had a sustained benefit post-transplantation on prolonging BOS-free survival and preserving graft function.

Limitations of this report include the retrospective design, being a single-centre study and the size of the study population. However, the study population reflects an average patient population in a lung transplant centre, and we believe the conclusions are both valid and important.

In conclusion, long-term treatment with azithromycin initially improves lung function then slows down the progression of BOS, although the effect of TLI may have affected the observed attenuation of FEV_1_ decline. Importantly, BOS progression and long-term survival are not affected by randomisation to placebo for a 3-month period, given the early cross-over to azithromycin and possibly due to TLI in case of further progression. Survival was not different between early or late onset of BOS and was better than many previously reported series.^14 15^
